# *N*-(2-Chloro­phen­yl)-1-(4-chloro­phen­yl)formamido 3-(2-nitrophenyl)­propano­ate

**DOI:** 10.1107/S1600536812048726

**Published:** 2012-11-30

**Authors:** Lin-Lan Fan, Hui Wang, Jing Ma, Xiu-Xiao Shi

**Affiliations:** a Department of Laboratory Center for Medical Sciences, School of Basic Medical Sciences, Lanzhou University, Lanzhou 730000, Gansu Province, People’s Republic of China; b Jiuquan Institute for Food and Drug Control, Jiuquan 735000, Gansu Province, People’s Republic of China; c Institute of Medicinal Chemistry, School of Pharmacy, Lanzhou University, Lanzhou 730000, Gansu Province, People’s Republic of China

## Abstract

In the title hydroxamic acid derivative, C_22_H_16_Cl_2_N_2_O_5_, the nitro-substituted benzene ring forms dihedral angles of 26.95 (15) and 87.06 (15)°, with the 4-chloro- and 2-chloro-substituted benzene rings, respectively. The dihedral angle between the chloro-substituted benzene rings is 68.19 (13)°. The O atoms of the nitro group were refined as disordered over two sets of sites with equal occupancies. In the crystal, weak C—H⋯O(=C) hydrogen bonds link mol­ecules along [100].

## Related literature

For applications of hydroxamic acid derivatives, see: Noh *et al.* (2009 ▶); Zeng *et al.* (2003 ▶). For the synthesis, see: Ayyangark *et al.* (1986 ▶). For related structures, see: Zhang *et al.* (2012 ▶); Ma *et al.* (2012 ▶).
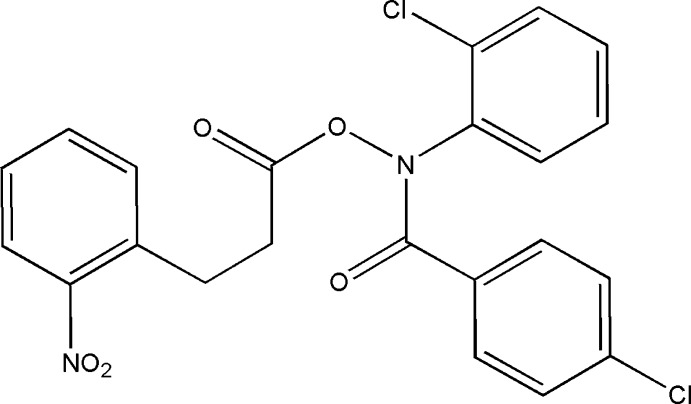


## Experimental

### 

#### Crystal data

C_22_H_16_Cl_2_N_2_O_5_
*M* *_r_* = 459.27
Triclinic, 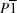

*a* = 9.1574 (8) Å
*b* = 10.1976 (6) Å
*c* = 12.1736 (8) Å
α = 91.847 (5)°
β = 108.327 (8)°
γ = 100.285 (6)°
*V* = 1057.06 (13) Å^3^
*Z* = 2
Mo *K*α radiation
μ = 0.34 mm^−1^
*T* = 293 K
0.32 × 0.28 × 0.25 mm

#### Data collection

Agilent SuperNova (Dual, Cu at zero, Eos) diffractometer
Absorption correction: multi-scan (*CrysAlis PRO*; Agilent, 2011 ▶) *T*_min_ = 0.843, *T*_max_ = 1.000
7898 measured reflections
4785 independent reflections
3434 reflections with *I* > 2σ(*I*)
*R*_int_ = 0.018

#### Refinement

*R*[*F* ^2^ > 2σ(*F* ^2^)] = 0.055
*wR*(*F* ^2^) = 0.139
*S* = 1.04
4785 reflections
298 parameters
24 restraints
H-atom parameters constrained
Δρ_max_ = 0.48 e Å^−3^
Δρ_min_ = −0.45 e Å^−3^

### 

Data collection: *CrysAlis PRO* (Agilent, 2011 ▶); cell refinement: *CrysAlis PRO*; data reduction: *CrysAlis PRO*; program(s) used to solve structure: *SHELXS97* (Sheldrick, 2008 ▶); program(s) used to refine structure: *SHELXL97* (Sheldrick, 2008 ▶); molecular graphics: *PLATON* (Spek, 2009 ▶); software used to prepare material for publication: *SHELXL97*.

## Supplementary Material

Crystal structure: contains datablock(s) global, I. DOI: 10.1107/S1600536812048726/lh5558sup1.cif

Structure factors: contains datablock(s) I. DOI: 10.1107/S1600536812048726/lh5558Isup2.hkl

Supplementary material file. DOI: 10.1107/S1600536812048726/lh5558Isup3.cml

Additional supplementary materials:  crystallographic information; 3D view; checkCIF report

## Figures and Tables

**Table 1:** Hydrogen-bond geometry (Å, °)

*D*—H⋯*A*	*D*—H	H⋯*A*	*D*⋯*A*	*D*—H⋯*A*
C11—H11⋯O1^i^	0.93	2.52	3.354 (4)	150
C13—H13⋯O3^ii^	0.93	2.48	3.223 (4)	137

Symmetry codes: (i) 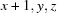; (ii) 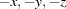.

**Fig. 1.**
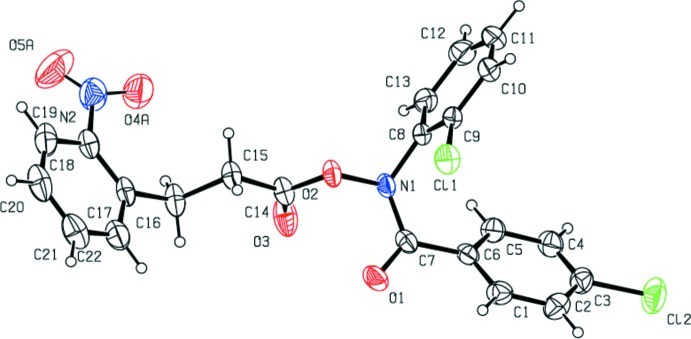
The molecular structure of the title compound with 30% probability displacement dllipsoids. H atoms are shown as small spheres of arbitrary radius. The disorder is not shown.

**Fig. 2.**
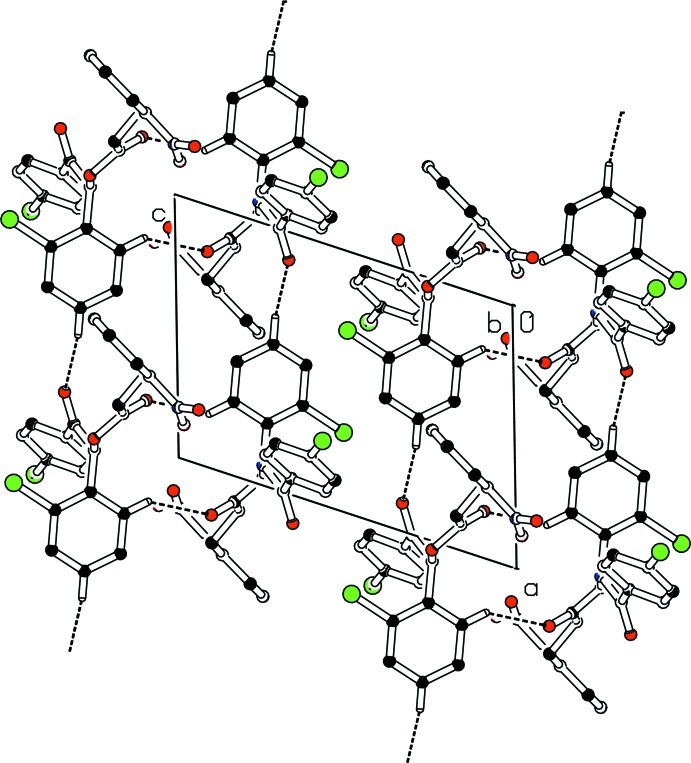
Part of the crystal structure with hydrogen bonds shown as dashed lines. The disorder is not shown.

## supplementary crystallographic information

### Comment

Hydroxamic acid derivatives have received considerable attention in recent years as the result of the discovery of their role in the biochemical toxicology of many drugs and other chemicals (Noh *et al.*, 2009; Zeng *et al.*, 2003). We have performed the crystal structure determination of the title hydroxamic acid derivative.

The molecular structure of the title compound is shown in Fig. 1. The nitro-substituted benzene ring (C17-C22) forms dihedral angles of 26.95 (15) and 87.06 (15)°, with the p-chloro (C1-C6) and o-chloro-substituted (C8-C13) benzene rings, respectively. The dihedral angle between the two chloro-substituted benzene rings is 68.19 (13) °. Closely related structures appear in the literature (Zhang *et al.*, 2012; Ma *et al.*, 2012). In the crystal, weak C—H···O(═C) hydrogen bonds links molecules along [100] (Fig. 2).

### Experimental

The title compound (I) was prepared according to the method described by Ayyangark *et al.* (1986). Crystals of (I) suitable for single-crystal X-ray analysis were grown by slow evaporation of a solution of (I) in dichloromethane-methanol (1:3 *v*/*v*).

### Refinement

Hydrogen atoms were placed in calculated positions with C—H = 0.93 and 0.97Å and included in a riding-model approximation with U_iso_(H) = 1.2U_eq_(C). The O atoms of the nitro group were refined as disorderd over two sets of sites (O4A,O5A/O4B,O5B) with equal occupancies. No geometric constraints were applied to the N—O distances or O—N—O angles as this had a negative effect on the refinment. The O atoms were restrained to be isotropic in nature, using ISOR 0.01 0.02 O4B O5A O5A O4A in SHELXL (Sheldrick, 2008).

### Crystal data

**Table:**

C_22_H_16_Cl_2_N_2_O_5_	*Z* = 2
*M**_r_* = 459.27	*F*(000) = 472
Triclinic, *P*1	*D*_x_ = 1.443 Mg m^−^^3^
*a* = 9.1574 (8) Å	Mo *K*α radiation, λ = 0.7107 Å
*b* = 10.1976 (6) Å	Cell parameters from 2906 reflections
*c* = 12.1736 (8) Å	θ = 3.0–28.5°
α = 91.847 (5)°	µ = 0.34 mm^−^^1^
β = 108.327 (8)°	*T* = 293 K
γ = 100.285 (6)°	Block, colourless
*V* = 1057.06 (13) Å^3^	0.32 × 0.28 × 0.25 mm

### Data collection

**Table:**

Agilent SuperNova (Dual, Cu at zero, Eos) diffractometer	4785 independent reflections
Radiation source: SuperNova (Mo) X-ray Source	3434 reflections with *I* > 2σ(*I*)
Mirror monochromator	*R*_int_ = 0.018
Detector resolution: 16.0733 pixels mm^-1^	θ_max_ = 28.6°, θ_min_ = 3.0°
ω scans	*h* = −12→12
Absorption correction: multi-scan (*CrysAlis PRO*; Agilent, 2011)	*k* = −11→13
*T*_min_ = 0.843, *T*_max_ = 1.000	*l* = −12→15
7898 measured reflections	

### Refinement

**Table:**

Refinement on *F*^2^	24 restraints
Least-squares matrix: full	H-atom parameters constrained
*R*[*F*^2^ > 2σ(*F*^2^)] = 0.055	*w* = 1/[σ^2^(*F*_o_^2^) + (0.0447*P*)^2^ + 0.5564*P*] where *P* = (*F*_o_^2^ + 2*F*_c_^2^)/3
*wR*(*F*^2^) = 0.139	(Δ/σ)_max_ < 0.001
*S* = 1.04	Δρ_max_ = 0.48 e Å^−^^3^
4785 reflections	Δρ_min_ = −0.45 e Å^−^^3^
298 parameters	

### Special details

**Table:**

Geometry. All e.s.d.'s (except the e.s.d. in the dihedral angle between two l.s. planes) are estimated using the full covariance matrix. The cell e.s.d.'s are taken into account individually in the estimation of e.s.d.'s in distances, angles and torsion angles; correlations between e.s.d.'s in cell parameters are only used when they are defined by crystal symmetry. An approximate (isotropic) treatment of cell e.s.d.'s is used for estimating e.s.d.'s involving l.s. planes.
Refinement. Refinement of *F*^2^ against ALL reflections. The weighted *R*-factor *wR* and goodness of fit *S* are based on *F*^2^, conventional *R*-factors *R* are based on *F*, with *F* set to zero for negative *F*^2^. The threshold expression of *F*^2^ > σ(*F*^2^) is used only for calculating *R*-factors(gt) *etc*. and is not relevant to the choice of reflections for refinement. *R*-factors based on *F*^2^ are statistically about twice as large as those based on *F*, and *R*- factors based on ALL data will be even larger.

### Fractional atomic coordinates and isotropic or equivalent isotropic displacement parameters (Å^2^)

**Table:**

	*x*	*y*	*z*	*U*_iso_*/*U*_eq_	Occ. (<1)
Cl1	0.30250 (8)	0.14141 (7)	0.49104 (6)	0.0635 (2)	
Cl2	0.24622 (12)	−0.53403 (8)	0.43213 (9)	0.0942 (3)	
O2	0.03359 (18)	0.18128 (15)	0.24969 (15)	0.0496 (4)	
O1	−0.1073 (2)	−0.01016 (18)	0.33664 (18)	0.0641 (5)	
O3	−0.1782 (3)	0.0981 (2)	0.09403 (19)	0.0827 (7)	
N1	0.0768 (2)	0.05360 (18)	0.25036 (18)	0.0455 (5)	
C9	0.3548 (3)	0.1106 (2)	0.3701 (2)	0.0473 (5)	
C8	0.2407 (3)	0.0716 (2)	0.2637 (2)	0.0436 (5)	
C7	0.0030 (3)	−0.0337 (2)	0.3102 (2)	0.0468 (5)	
C6	0.0626 (3)	−0.1603 (2)	0.3349 (2)	0.0456 (5)	
C3	0.1726 (3)	−0.3910 (2)	0.3938 (3)	0.0583 (7)	
C14	−0.0991 (3)	0.1893 (3)	0.1623 (2)	0.0532 (6)	
C13	0.2822 (3)	0.0474 (3)	0.1654 (3)	0.0576 (7)	
H13	0.2058	0.0246	0.0927	0.069*	
C4	0.1748 (3)	−0.3425 (2)	0.2910 (3)	0.0600 (7)	
H4	0.2127	−0.3874	0.2414	0.072*	
C17	−0.3174 (3)	0.4808 (3)	0.1221 (2)	0.0558 (7)	
C1	0.0565 (3)	−0.2147 (3)	0.4362 (2)	0.0565 (6)	
H1	0.0148	−0.1728	0.4849	0.068*	
C5	0.1203 (3)	−0.2260 (2)	0.2610 (2)	0.0538 (6)	
H5	0.1223	−0.1919	0.1915	0.065*	
C18	−0.2762 (3)	0.6005 (3)	0.0793 (2)	0.0594 (7)	
C15	−0.1275 (3)	0.3281 (2)	0.1738 (2)	0.0545 (6)	
H15A	−0.0445	0.3904	0.1583	0.065*	
H15B	−0.1223	0.3501	0.2532	0.065*	
N2	−0.1934 (4)	0.6103 (4)	−0.0050 (3)	0.0862 (8)	
C11	0.5514 (4)	0.0928 (3)	0.2855 (4)	0.0758 (9)	
H11	0.6565	0.0978	0.2930	0.091*	
C12	0.4391 (4)	0.0577 (3)	0.1778 (3)	0.0731 (9)	
H12	0.4689	0.0409	0.1132	0.088*	
C10	0.5115 (3)	0.1204 (3)	0.3813 (3)	0.0634 (7)	
H10	0.5887	0.1455	0.4533	0.076*	
C2	0.1111 (3)	−0.3299 (3)	0.4659 (3)	0.0642 (7)	
H2	0.1064	−0.3660	0.5342	0.077*	
C16	−0.2842 (3)	0.3464 (3)	0.0924 (3)	0.0670 (8)	
H16A	−0.2836	0.3403	0.0129	0.080*	
H16B	−0.3667	0.2756	0.0986	0.080*	
C20	−0.3816 (4)	0.7203 (4)	0.1959 (3)	0.0823 (10)	
H20	−0.4026	0.7998	0.2210	0.099*	
C22	−0.3929 (4)	0.4867 (3)	0.2037 (3)	0.0744 (8)	
H22	−0.4229	0.4084	0.2350	0.089*	
C21	−0.4252 (4)	0.6038 (4)	0.2402 (3)	0.0854 (10)	
H21	−0.4768	0.6038	0.2949	0.102*	
C19	−0.3082 (4)	0.7198 (3)	0.1157 (3)	0.0744 (9)	
H19	−0.2790	0.7987	0.0850	0.089*	
O4B	−0.2146 (9)	0.5291 (11)	−0.0774 (8)	0.137 (4)	0.50
O5A	−0.2098 (13)	0.6859 (11)	−0.0663 (10)	0.171 (4)	0.50
O4A	−0.1345 (10)	0.5136 (9)	−0.0205 (7)	0.114 (3)	0.50
O5B	−0.0987 (8)	0.7288 (7)	−0.0029 (5)	0.0943 (17)	0.50

### Atomic displacement parameters (Å^2^)

**Table:**

	*U*^11^	*U*^22^	*U*^33^	*U*^12^	*U*^13^	*U*^23^
Cl1	0.0684 (4)	0.0682 (4)	0.0530 (4)	0.0221 (3)	0.0142 (3)	0.0018 (3)
Cl2	0.1052 (7)	0.0560 (4)	0.1179 (8)	0.0351 (4)	0.0201 (6)	0.0158 (5)
O2	0.0434 (9)	0.0434 (8)	0.0577 (10)	0.0202 (7)	0.0046 (7)	0.0009 (7)
O1	0.0492 (10)	0.0645 (11)	0.0909 (14)	0.0216 (9)	0.0346 (10)	0.0043 (10)
O3	0.0803 (14)	0.0757 (13)	0.0724 (14)	0.0383 (11)	−0.0127 (11)	−0.0204 (11)
N1	0.0394 (10)	0.0423 (10)	0.0578 (12)	0.0189 (8)	0.0140 (9)	0.0052 (9)
C9	0.0416 (12)	0.0391 (11)	0.0630 (15)	0.0134 (9)	0.0159 (11)	0.0125 (11)
C8	0.0388 (12)	0.0397 (11)	0.0571 (14)	0.0161 (9)	0.0171 (10)	0.0110 (10)
C7	0.0368 (12)	0.0475 (12)	0.0557 (14)	0.0117 (10)	0.0133 (10)	−0.0034 (11)
C6	0.0340 (11)	0.0407 (11)	0.0608 (15)	0.0053 (9)	0.0154 (10)	−0.0026 (10)
C3	0.0549 (15)	0.0398 (12)	0.0733 (19)	0.0091 (11)	0.0117 (13)	0.0022 (12)
C14	0.0477 (14)	0.0620 (15)	0.0497 (14)	0.0255 (12)	0.0079 (11)	0.0009 (12)
C13	0.0608 (16)	0.0535 (14)	0.0696 (18)	0.0201 (12)	0.0311 (14)	0.0147 (13)
C4	0.0606 (16)	0.0457 (13)	0.080 (2)	0.0152 (12)	0.0298 (14)	−0.0048 (13)
C17	0.0432 (13)	0.0602 (15)	0.0573 (15)	0.0255 (12)	−0.0009 (11)	0.0024 (12)
C1	0.0568 (15)	0.0526 (14)	0.0655 (17)	0.0093 (12)	0.0289 (13)	0.0018 (12)
C5	0.0560 (15)	0.0455 (13)	0.0635 (16)	0.0116 (11)	0.0243 (12)	0.0003 (11)
C18	0.0480 (14)	0.0698 (17)	0.0559 (16)	0.0250 (13)	0.0032 (12)	0.0071 (13)
C15	0.0506 (14)	0.0529 (14)	0.0571 (15)	0.0240 (11)	0.0064 (11)	0.0015 (12)
N2	0.088 (2)	0.106 (2)	0.071 (2)	0.037 (2)	0.0235 (16)	0.026 (2)
C11	0.0516 (17)	0.0695 (18)	0.122 (3)	0.0229 (14)	0.0430 (19)	0.0298 (19)
C12	0.086 (2)	0.0659 (18)	0.098 (3)	0.0309 (16)	0.063 (2)	0.0240 (17)
C10	0.0421 (14)	0.0575 (15)	0.089 (2)	0.0134 (12)	0.0159 (14)	0.0201 (15)
C2	0.0716 (18)	0.0520 (15)	0.0662 (18)	0.0074 (13)	0.0209 (15)	0.0079 (13)
C16	0.0606 (17)	0.0610 (16)	0.0701 (18)	0.0311 (13)	−0.0015 (14)	0.0003 (14)
C20	0.070 (2)	0.080 (2)	0.089 (2)	0.0403 (18)	0.0030 (18)	−0.0141 (19)
C22	0.0614 (18)	0.084 (2)	0.084 (2)	0.0323 (16)	0.0222 (16)	0.0157 (17)
C21	0.070 (2)	0.111 (3)	0.083 (2)	0.045 (2)	0.0217 (18)	−0.001 (2)
C19	0.0672 (19)	0.0629 (17)	0.082 (2)	0.0270 (15)	0.0005 (16)	0.0067 (16)
O4B	0.106 (6)	0.178 (8)	0.124 (7)	−0.001 (5)	0.055 (5)	−0.074 (6)
O5A	0.222 (9)	0.153 (7)	0.193 (8)	0.065 (7)	0.119 (7)	0.114 (7)
O4A	0.140 (7)	0.131 (5)	0.124 (6)	0.071 (5)	0.087 (5)	0.062 (5)
O5B	0.111 (5)	0.096 (4)	0.082 (4)	0.012 (3)	0.042 (3)	0.027 (3)

### Geometric parameters (Å, º)

**Table:**

Cl1—C9	1.721 (3)	C5—H5	0.9300
Cl2—C3	1.731 (3)	C18—N2	1.451 (4)
O2—N1	1.426 (2)	C18—C19	1.391 (4)
O2—C14	1.357 (3)	C15—H15A	0.9700
O1—C7	1.211 (3)	C15—H15B	0.9700
O3—C14	1.189 (3)	C15—C16	1.513 (3)
N1—C8	1.436 (3)	N2—O4B	1.135 (8)
N1—C7	1.385 (3)	N2—O5A	1.087 (8)
C9—C8	1.377 (3)	N2—O4A	1.241 (8)
C9—C10	1.383 (3)	N2—O5B	1.350 (7)
C8—C13	1.392 (4)	C11—H11	0.9300
C7—C6	1.492 (3)	C11—C12	1.377 (5)
C6—C1	1.381 (4)	C11—C10	1.363 (4)
C6—C5	1.387 (3)	C12—H12	0.9300
C3—C4	1.365 (4)	C10—H10	0.9300
C3—C2	1.370 (4)	C2—H2	0.9300
C14—C15	1.494 (3)	C16—H16A	0.9700
C13—H13	0.9300	C16—H16B	0.9700
C13—C12	1.380 (4)	C20—H20	0.9300
C4—H4	0.9300	C20—C21	1.367 (5)
C4—C5	1.385 (3)	C20—C19	1.348 (5)
C17—C18	1.380 (4)	C22—H22	0.9300
C17—C16	1.512 (3)	C22—C21	1.373 (4)
C17—C22	1.383 (4)	C21—H21	0.9300
C1—H1	0.9300	C19—H19	0.9300
C1—C2	1.372 (4)		
			
C14—O2—N1	114.19 (17)	H15A—C15—H15B	107.7
O2—N1—C8	109.22 (16)	C16—C15—H15A	108.9
C7—N1—O2	112.21 (17)	C16—C15—H15B	108.9
C7—N1—C8	123.88 (18)	O4B—N2—C18	123.7 (6)
C8—C9—Cl1	120.05 (18)	O4B—N2—O5B	119.1 (6)
C8—C9—C10	120.3 (3)	O5A—N2—C18	119.8 (6)
C10—C9—Cl1	119.6 (2)	O5A—N2—O4B	91.5 (9)
C9—C8—N1	121.6 (2)	O5A—N2—O4A	121.4 (7)
C9—C8—C13	120.4 (2)	O5A—N2—O5B	50.8 (6)
C13—C8—N1	118.0 (2)	O4A—N2—C18	116.9 (4)
O1—C7—N1	121.4 (2)	O4A—N2—O5B	112.4 (6)
O1—C7—C6	122.4 (2)	O5B—N2—C18	116.9 (4)
N1—C7—C6	116.12 (19)	C12—C11—H11	119.4
C1—C6—C7	117.4 (2)	C10—C11—H11	119.4
C1—C6—C5	119.0 (2)	C10—C11—C12	121.3 (3)
C5—C6—C7	123.5 (2)	C13—C12—H12	119.9
C4—C3—Cl2	119.1 (2)	C11—C12—C13	120.2 (3)
C4—C3—C2	121.3 (2)	C11—C12—H12	119.9
C2—C3—Cl2	119.6 (2)	C9—C10—H10	120.4
O2—C14—C15	107.5 (2)	C11—C10—C9	119.1 (3)
O3—C14—O2	124.2 (2)	C11—C10—H10	120.4
O3—C14—C15	128.2 (2)	C3—C2—C1	119.2 (3)
C8—C13—H13	120.6	C3—C2—H2	120.4
C12—C13—C8	118.7 (3)	C1—C2—H2	120.4
C12—C13—H13	120.6	C17—C16—C15	110.6 (2)
C3—C4—H4	120.2	C17—C16—H16A	109.5
C3—C4—C5	119.5 (2)	C17—C16—H16B	109.5
C5—C4—H4	120.2	C15—C16—H16A	109.5
C18—C17—C16	127.2 (3)	C15—C16—H16B	109.5
C18—C17—C22	115.7 (3)	H16A—C16—H16B	108.1
C22—C17—C16	117.1 (3)	C21—C20—H20	120.0
C6—C1—H1	119.5	C19—C20—H20	120.0
C2—C1—C6	120.9 (2)	C19—C20—C21	120.0 (3)
C2—C1—H1	119.5	C17—C22—H22	118.8
C6—C5—H5	120.0	C21—C22—C17	122.4 (3)
C4—C5—C6	119.9 (3)	C21—C22—H22	118.8
C4—C5—H5	120.0	C20—C21—C22	119.9 (3)
C17—C18—N2	121.9 (3)	C20—C21—H21	120.0
C17—C18—C19	122.4 (3)	C22—C21—H21	120.0
C19—C18—N2	115.7 (3)	C18—C19—H19	120.2
C14—C15—H15A	108.9	C20—C19—C18	119.6 (3)
C14—C15—H15B	108.9	C20—C19—H19	120.2
C14—C15—C16	113.4 (2)		
			
Cl1—C9—C8—N1	−1.0 (3)	C14—C15—C16—C17	170.5 (3)
Cl1—C9—C8—C13	179.59 (17)	C4—C3—C2—C1	−2.6 (4)
Cl1—C9—C10—C11	178.5 (2)	C17—C18—N2—O4B	36.3 (8)
Cl2—C3—C4—C5	−178.2 (2)	C17—C18—N2—O5A	151.0 (9)
Cl2—C3—C2—C1	178.4 (2)	C17—C18—N2—O4A	−13.5 (7)
O2—N1—C8—C9	78.1 (2)	C17—C18—N2—O5B	−150.8 (4)
O2—N1—C8—C13	−102.5 (2)	C17—C18—C19—C20	0.2 (4)
O2—N1—C7—O1	13.8 (3)	C17—C22—C21—C20	−0.4 (5)
O2—N1—C7—C6	−168.96 (18)	C1—C6—C5—C4	−1.8 (4)
O2—C14—C15—C16	−171.8 (2)	C5—C6—C1—C2	2.1 (4)
O1—C7—C6—C1	−34.9 (3)	C18—C17—C16—C15	89.3 (3)
O1—C7—C6—C5	143.9 (3)	C18—C17—C22—C21	0.0 (4)
O3—C14—C15—C16	5.3 (5)	N2—C18—C19—C20	−178.5 (3)
N1—O2—C14—O3	2.9 (4)	C12—C11—C10—C9	1.1 (4)
N1—O2—C14—C15	−179.87 (19)	C10—C9—C8—N1	176.6 (2)
N1—C8—C13—C12	−176.8 (2)	C10—C9—C8—C13	−2.7 (3)
N1—C7—C6—C1	147.9 (2)	C10—C11—C12—C13	−1.2 (4)
N1—C7—C6—C5	−33.3 (3)	C2—C3—C4—C5	2.9 (4)
C9—C8—C13—C12	2.6 (3)	C16—C17—C18—N2	0.9 (4)
C8—N1—C7—O1	148.4 (2)	C16—C17—C18—C19	−177.7 (2)
C8—N1—C7—C6	−34.3 (3)	C16—C17—C22—C21	178.1 (3)
C8—C9—C10—C11	0.8 (4)	C22—C17—C18—N2	178.7 (3)
C8—C13—C12—C11	−0.7 (4)	C22—C17—C18—C19	0.1 (4)
C7—N1—C8—C9	−57.7 (3)	C22—C17—C16—C15	−88.4 (3)
C7—N1—C8—C13	121.7 (2)	C21—C20—C19—C18	−0.5 (5)
C7—C6—C1—C2	−179.0 (2)	C19—C18—N2—O4B	−145.0 (8)
C7—C6—C5—C4	179.4 (2)	C19—C18—N2—O5A	−30.3 (10)
C6—C1—C2—C3	0.1 (4)	C19—C18—N2—O4A	165.2 (6)
C3—C4—C5—C6	−0.6 (4)	C19—C18—N2—O5B	27.9 (5)
C14—O2—N1—C8	132.4 (2)	C19—C20—C21—C22	0.6 (5)
C14—O2—N1—C7	−86.4 (2)		

### Hydrogen-bond geometry (Å, º)

**Table:**

*D*—H···*A*	*D*—H	H···*A*	*D*···*A*	*D*—H···*A*
C11—H11···O1^i^	0.93	2.52	3.354 (4)	150
C13—H13···O3^ii^	0.93	2.48	3.223 (4)	137

Symmetry codes: (i) *x*+1, *y*, *z*; (ii) −*x*, −*y*, −*z*.
